# Management of X-linked adrenoleukodystrophy in Morocco: actual situation

**DOI:** 10.1186/s13104-017-2902-4

**Published:** 2017-11-07

**Authors:** F. Z. Madani Benjelloun, Y. Kriouile, D. Cheillan, H. Daoud-Tetouani, L. Chabraoui

**Affiliations:** 1 Biochemistry Laboratory, Faculty of Medicine and Pharmacy, University Mohammed V Souissi, Rabat, Morocco; 2 Neuropediatric Department, Children’s Hospital, Rabat, Morocco; 3 Department of Inherited and Metabolic Diseases and Neonatal Screening, Hospices Civils , Lyon, France; 4 Department of Biomedical Engineering, Bunker Hill Community College, Boston, USA; 5 Central Laboratory of Inherited and Metabolic Diseases, Ibn Sina Hospital, Rabat, Morocco

**Keywords:** X-ALD, X-linked Adrenoleukodystrophy, Neurodegenerative disorder, Rare disease, Mutation, ABCD1 gene, Metabolic disease

## Abstract

**Objectives:**

X-linked adrenoleukodystrophy is a neurodegenerative disorder caused by mutations in the *ABCD1* gene. Adrenomyeloneuropathy and childhood cerebral Adrenoleukodystrophy are the most common phenotypes. This paper focuses on a descriptive study of the first program of diagnosis, treatment, and follow-up of this disease in Morocco.

**Results:**

We developed three protocols of X-linked Adrenoleukodystrophy management: general protocol, asymptomatic protocol, and heterozygous protocol. Over a period of 5 years, we recruited eight families with 16 patients. Clinically, the presentation is primary adrenal insufficiency and behavioral changes. All patients had elevated levels of very long fatty acids. This is the first study of X-linked adrenoleukodystrophy in Morocco. It shows the importance of this metabolic disease and broadens perspectives in terms of its diagnosis and its treatment.

## Introduction

X-linked adrenoleukodystrophy is a rare neurodegenerative disease, and it is the most common peroxisomal disease. It is caused by *ABCD1* gene mutations [1] and inherited as an X-Linked trait [2]. Clinically, there are various phenotypes associated with *ABCD1* gene mutations. We consider principally cerebral childhood form (CCALD), which is characterized by neurological symptoms associated or unassociated with adrenal insufficiency, adolescent cerebral form, adult cerebral form, Adrenomyeloneuropathy (AMN), Addison disease form, and asymptomatic forms [3]. One of the characteristics of this inherited disease is the lack of phenotype–genotype correlation [4–6].

This metabolic disease results in impaired β-oxidation of a very long chain fatty acids (VLCFA), which are plasma biomarkers useful for the diagnostic [7, 8]. Clinically, the diagnosis of X-ALD is very difficult [9] because the symptoms are similar to many neurometabolic diseases. Moreover, the disease usually begins with an asymptomatic stage before manifesting the first symptoms and no phenotype-genotype correlations, leading to diagnostic errors.

The prognosis of X-ALD is generally very poor: the patient may die within a few years or live with various health problems, but good general care can improve the prognosis, especially with early diagnosis. Also, X-ALD can have a devastating social and psychological impact on the patient and his nuclear and extended family [10].

Current treatment is based on Hematopoietic Stem Cells Transplantation (HSCT). However, other approaches are under evaluation [11].

In Morocco, there are no large studies describing the profile of X-ALD patients, and our project is the first large prospective study of X-ALD in Morocco. The study was conducted from 2013 to 2017. The objective is to better understand the disease in the local context and to propose solutions for patients and their families.

## Main text

### Materials and methods

Our program aims to install a management protocol of X-ALD in Moroccan medical structures and to inform the population about it. As a complicated disease with various phenotypes, the protocol must be as detailed as possible. To achieve this objective, we introduced the implicated medical structures in the following facilities: the Neuropediatric Department of The Children’s Hospital in Rabat, the Biochemical Laboratory of Medicine and Pharmacy Faculty of Rabat, and the Hereditary Central Laboratory. Furthermore, a multidisciplinary team was formed including neurologists, neuro-pediatricians, pediatricians, biologists, endocrinologists, specialists on bio-informatics and dieticians.

The first step in drawing up the program was the collection of scientific data on recent studies and scientific progress on the disease, to ensure a perfect program that corresponds to actual findings. The multidisciplinary team was contacted and formed simultaneously with asking for collaborations and conventions between different structures. Then we prepared the written material that was subsequently used for different protocols. The program included inclusion criteria and three protocols corresponding to different forms of the disease with complete details about diagnosis, follow-up, and treatment for each form. After ethics approval, we began the execution of the program.

Patients were recruited from the Neuropediatric Department of The Children’s Hospital of Rabat, which receives patients from all regions of Morocco. Whenever a suspect case is identified, we start the corresponding protocol.

We included an awareness-raising component for medical personnel to increase their familiarity with the disease and avoid diagnostic errors. For non-medical personnel, the objective was to enable them to recognize the first signs of the disease and familiarize them with the qualified medical structures for X-ALD. This would be accomplished by organizing conferences and meetings around X-ALD in medical centers, societies, and schools, and by participating in scientific events and conferences relevant to the disease.

### Results

We established a list of criteria for inclusion in our program. Patients were included in four cases: (1) Boys with neurological symptoms (pyramidal weakness/spasticity, ataxia, gait disturbance, convulsions, dementia, difficulty in understanding spoken language, worsening handwriting, incoordination), associated to Vision and hearing disturbance or behavioral symptoms. (2) All males with primary adrenocortical insufficiency, with or without evidence of neurologic abnormality. (3) Women with progressive paraparesis, abnormalities of sphincter control, and sensory disturbances mainly affecting the legs with or without neurologic abnormality. (4) All patients belonging to a family with a history of X-ALD or presenting demyelinating lesions in magnetic resonance imaging (MRI) (Table 1).

**Table 1:** Criteria of recruitment of patients in X-ALD program

Neurological symptoms	Pyramidal weakness/spasticity Ataxia Gait disturbance Convulsions Dementia,
General symptoms	Vision and hearing disturbance
Adrenal symptoms	Skin pigmentation Vomiting Diarrhea Anorexia
Intellectual and behavioral changes	Hyperactivity Nervousness Attention deficits Isolation
School performance	Lack of concentration
MRI finding	Abnormalities in cerebral MRI Demyelinating lesions
Others	Belonging to a family with a history of X-ALD

We developed three protocols for each type of patient: index case, asymptomatic case, and heterozygous women.

**Fig. 1:**
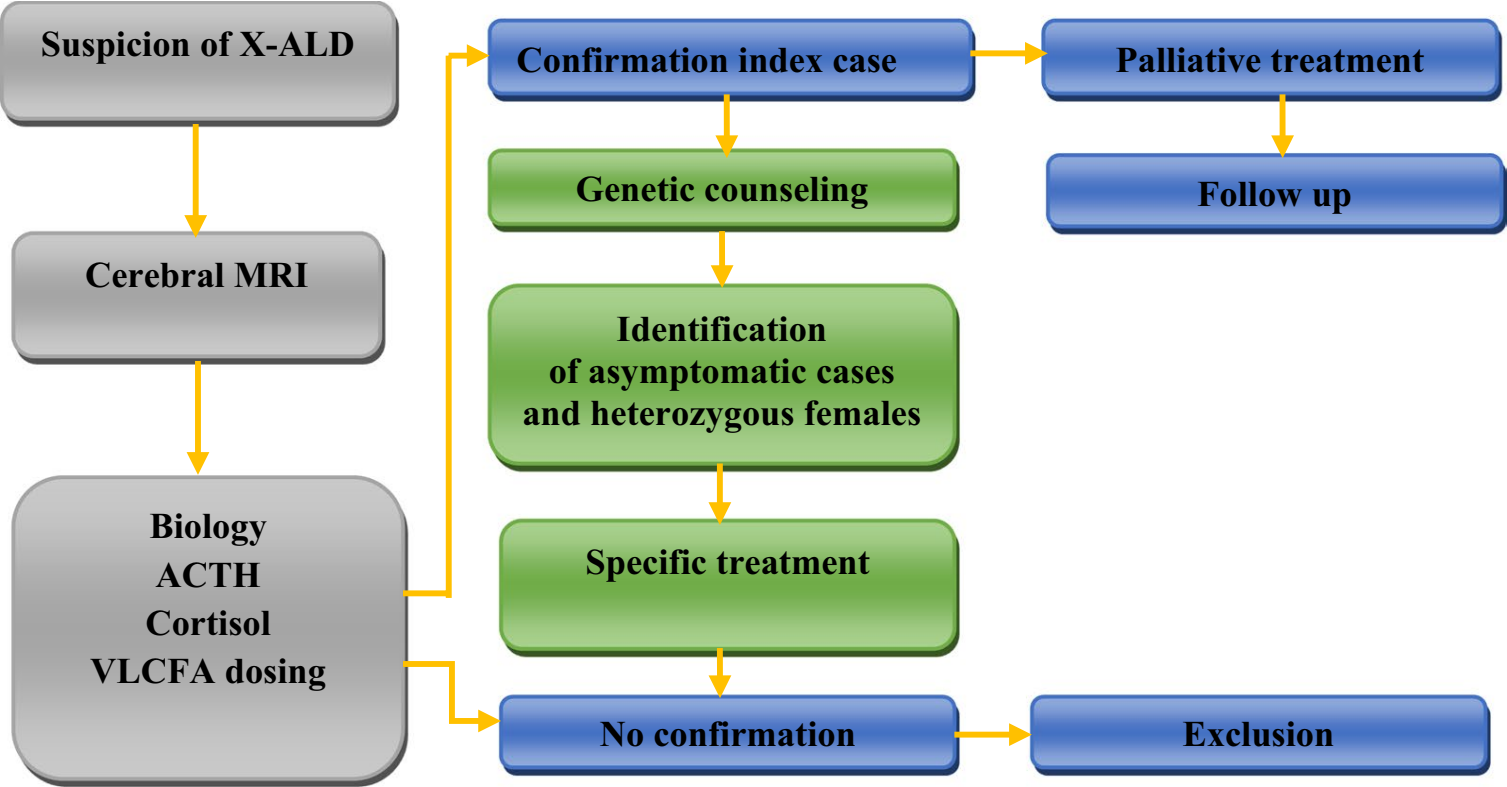
Diagram of X-linked adrenoleukodystrophy diagnosing and follow up. *X*-*ALD* X-linked adrenoleukodystrophy, *MRI* magnetic resonance imaging, *VLCFA* very long chain fatty acids

When a suspected case was identified, the diagnosis of X-ALD was made basing on clinical manifestations, cerebral MRI, general biologic assessment, cortisol and ACTH levels. Plasma VLCFAs levels were determined by capillary gas chromatography-mass spectrometry, including lignoceric acid (C24:0), pentacosanoic acid (C25:0), and hexacosanoic acid (C26:0), and their ratios to behenic acid (C22:0) were used to confirm the diagnosis of X-ALD [12]. DNA analysis of the *ABCD1* gene was performed after obtaining informed consent from parents. Consent forms were written in Arabic and French language to ensure that patients understood them. After confirmation of index case X-ALD, we scheduled a genetic consultation to explain the disease to parents and to inform them of the next steps. Then, a pedigree of the family was dressed, and family members were examined for VLCFA levels and *ABCD1* gene to identify asymptomatic cases and heterozygous females (Fig. 1). For index case, we established a specific treatment with quarterly follow-up or according to patient health status. A toll-free number was provided to patients to ensure continuous availability.

**Fig. 2:**
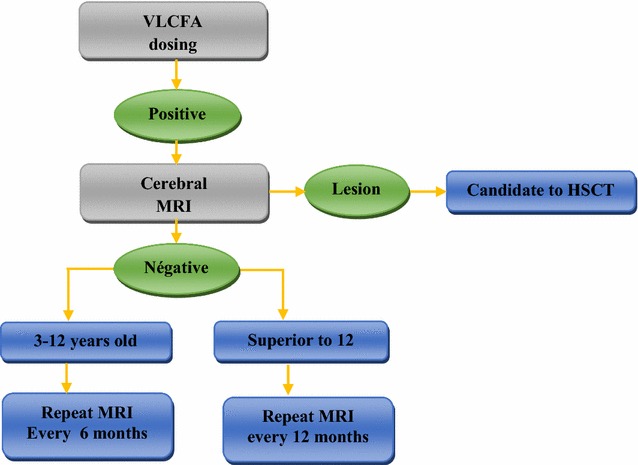
Diagram of asymptomatic boys follow-up. *MRI* magnetic resonance imaging, *HSCT* hematopoietic stem cells transplantation

For asymptomatic cases, and after confirmation of X-ALD in genetic counseling, asymptomatic children were evaluated every 6 months with MRI, from the age of 4–12 years, to follow demyelinating lesions. After 12 years of age, patients were evaluated with IRM every 12 months. If cerebral lesions were identified, the patient was transferred to the Hematopoietic Stem Cells Transplantation (HSCT) department (Fig. 2).

**Fig. 3:**
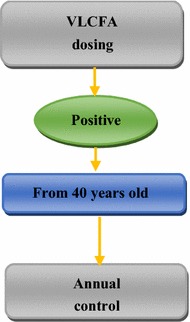
Diagram of heterozygous females follow-up. *VLCFA* very long chain fatty acids

For heterozygous females, after confirmation, we explained the risk of disease transmission to offspring and prevention methods, particularly prenatal diagnosis. Patients over 40 years of age were followed up with annually to detect the first symptoms of eventual AMN and provide adequate treatment (Fig. 3).

To this end, we organized 25 events in hospitals, schools, societies, and universities. Beneficiaries of awareness-raising activities included medical personnel, parents of patients and teachers in schools. We prepared awareness notices that were distributed during events. Since X-ALD is a costly disease for families, we collaborated with three international centers that offer free analysis.

To date, fifteen families were included in the program, with 39-suspected patients that manifested disease symptoms. No case had a family history of X-ALD. In two cases, patients with an adrenal sign were included, whereas all other patients exhibited neuropsychiatric symptoms. After confirmation, eight families were retained, with 16 patients, including 4 heterozygous females and 12 children.

Females were between 26 and 42 years old, all asymptomatic. Boys were between 4 and 8 years old, 4 asymptomatic and 8 cerebral child form (CCALD) confirmed by specific demyelinating lesions in MRI and high levels of VLCFA and their ratios. All asymptomatic patients were discovered in genetic counseling. The boys presented neurologic symptoms (hyperactivity, Isolation, paraparesis, gait disturbance), behavioral changes (Diminution of school performances, loss of concentration) and symptoms of adrenal insufficiency (Skin pigmentation, digestive symptoms) (Table 2).

**Table 2:** Profile of Moroccan patients with X-ALD

Case no	Age at onset	Initial symptom	Age at dignosis (interval)	Phenotype
1	–	–	25–30	Asymptomatic
2	6	Hyperactivity	5–10	Asymptomatic
3	–	–	25–30	Asymptomatic
4	7	Isolation	5–10	CCALD
5	7	Diminution of school performances	5–10	CCALD
6	5	–	5–10	Asymptomatic
7	–	–	35–40	Asymptomatic
8	6	Behavioral changes	5–10	CCALD
9	4	–	3–5	Asymptomatic
10	2	Skin pigmentation	3–5	CCALD
11	5	Paraparesis	5–10	CCALD
12	6	Digestive symptoms	5–10	CCALD
13	6	Gait disturbance	5–10	CCALD
14	–	–	40–45	Asymptomatic
15	6	Gait disturbance	5–10	CCALD
16	–	–	10–15	Asymptomatic

### Discussion

We report 16 patients (69% males and 31% females) with X-ALD. Genetic counseling was of great importance because many asymptomatic patients can be discovered in this manner [13]. In this study, all heterozygous females and four children were identified following genetic counseling, representing 50% of the study population. The same percentage of asymptomatic individuals was found in a study from Japan [14]. Only 11% of patients were asymptomatic in a study from Spain [13], and 7% in a study from South America [15]. In a study from Canada, 65% of the individuals were asymptomatic, in a population of 48 patients with 69% males and 31% females [16], which considerably approximates our results.

This percentage of 50% asymptomatic patients is an advantage for our population of males as well as females, as long as patients are diagnosed in a very early stage of the disease to undergo HSCT for males. For females, it is of big importance because of the difficulty associated with diagnosing symptomatic females with multiple sclerosis until identification of an index male in the family [17].

Two patients had an adrenal insufficiency as a first sign (12%). Primary adrenal insufficiency, also known as Addison’s disease, is due to peroxisomal disorders including X-ALD in 5% of cases [18], which shows how early diagnosis can identify adrenal-only phenotypes in young people who may be at risk of developing other symptoms in later life. It also demonstrates the importance of raising awareness of X-ALD in Addison’s disease, which was included in our program.

The profile of the Moroccan population contains two phenotypes: asymptomatic and CCALD in boys with differences in first symptoms and clinical manifestations. There was no AMN profile. Females were all asymptomatic; nevertheless, Semmler et al. reported that approximately half of heterozygous females develop moderate spastic paresis resembling the AMN phenotype [19]. The cerebral form is very rare in them [20]. We estimate that X-ALD is under-diagnosed: many patients will wait nearly a decade for a definitive diagnosis and at least half of cases will remain unresolved [21]. Periodic follow up is very important in order to control the evolution of X-ALD and monitor changes in patient status because of the non-predictive aspect of X-ALD. In fact, a patient can pass from one phenotype to another [9]. Thus, treatment must be adapted to the new status.

By consulting the Children Hospital’s register for the period between 2008 and 2013, we found that just three cases of X-ALD were diagnosed over a period of 5 years. There was no evidence of genetic counseling or follow-up. In contrast, during our 5-years program, we identified 16 cases of X-ALD, for which genetic counseling was performed and a complete disease management program was in place. This demonstrates the importance of our program and its impact on the quality of disease management.

Our structure has also provided a continuous support service for parents of children, for which we have dedicated a 24-h toll-free number to assist parents in managing X-ALD crises and answer their requests for information. This service provided moral support for patients and their parents, which had a positive impact on their quality of life.

As a first complete program of X-ALD management in Morocco, and given the rare aspect of the disease, its implementation has taken a great time and effort to get started. It was difficult to collaborate with different medical structures in the beginning of the study.

Nowadays, the Moroccan population has better access to MRI [22] as well as to the different analysis required for disease management, which are provided to all patients free of charge. Furthermore, collaboration with different structures has led to better management of X-ALD at the national scale.

### Conclusion

This first study of X-linked adrenoleukodystrophy in Morocco, conducted for the first time in Morocco, shows the importance of this metabolic disease in our country and broadens perspectives in terms of its diagnosis and its treatment. These achievements have an important impact on X-ALD families’ quality of life as well as for all X-ALD patients, for whom we have a specific protocol for diagnosis, follow up and treatment. All investigations are freely given, and all X-ALD families included in our program understand the disease with all its aspects.

Hence, we expect to characterize the mutational profile of X-ALD in the Moroccan population, and to conduct investigations in prenatal diagnosis in the near future.

## Limitations

Due to the rare aspect of the disease, this study required a great effort and time to be implemented.

## Abbreviations

X-ALD: X-linked adrenoleukodystrophy
AMN: adrenomyeloneuropathy
CCALD: childhood cerebral ALD
VLCFA: very long chain fatty acids
MRI: magnetic resonance imaging
HSCT: hematopoietic stem cells transplantation
